# Characterization of functional sweetened condensed milk formulated with flavoring and sugar substitute

**DOI:** 10.1002/fsn3.2477

**Published:** 2021-07-21

**Authors:** Mohammad Jouki, Somayeh Jafari, Ali Jouki, Naimeh Khazaei

**Affiliations:** ^1^ Department of Food Science and Technology Faculty of Biological Sciences North Tehran Branch Islamic Azad University Tehran Iran; ^2^ Department of Chemistry Faculty of Science Shahid Chamran University of Ahvaz Ahvaz Iran

**Keywords:** cinnamon, flavored condensed milk, formulation, fructose, functional properties

## Abstract

In the present study, the effect of sugar replacement and enrichment with cinnamon extract (CE) on the physicochemical, functional, and organoleptic properties of sweetened condensed milk (SCM) and the production of flavored functional dairy dessert was investigated. The results demonstrated that by adding CE (0.5 and 1%) and fructose sugar (50 and 100% replacement) in the formulation containing maltodextrin, the physicochemical, functional, and organoleptic properties of SCM samples were improved. However, adding fructose and CE at the maximum concentration increased the acidity, particle size, redness (a), yellowness (b) and decreased the lightness (L), viscosity, and sensory scores of the SCM samples (*p* < .05). Scanning electron microscopy images demonstrated that as the fructose level increased, the number of cavities increased, while the size of the cavities decreased (*p* < .05). Microstructure analysis also showed that the application of CE increased the density of the structure in the SCM samples. However, the SCM samples formulated with maltodextrin, 0.5% CE, and 50% fructose substitution were identified as optimal samples. Evaluation of the functional properties of SCM formulated with 0.5% CE revealed that the total phenolic content (TPC) and DPPH radical scavenging activity were in the range of 139.21–143.24 mg GAE / g and 50.12%–52.01%, respectively.

## Graphical abstract

Blurb for etocSweetened condensed milk (SCM) is a dairy product obtained by evaporating some water from skim milk or whole milk with adding sugar. In the present study, the effect of sugar replacement and enrichment with cinnamon extract (CE) on the physicochemical, functional, and organoleptic properties of sweetened condensed milk (SCM) and the production of flavored functional dairy dessert was investigated. The results demonstrated that by adding CE (0.5 and 1%) and fructose sugar (50 and 100% replacement) in the formulation containing maltodextrin, the physicochemical, functional, and organoleptic properties of SCM samples were improved.

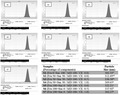

## 1 INTRODUCTION

Sweetened condensed milk (SCM) is a dairy product obtained by evaporating some water from skim milk or whole milk with adding sugar. This product is obtained by the heat treatment and the addition of sweeteners such as sucrose and can be consumed as a dessert (Silva, et al., 2020). SCM has consistency, light brown color, unique flavor and a lack of perceptible crystals in the mouth, and in terms of nutrition, has high levels of minerals, carbohydrates, and proteins (Silva, et al., 2020). Flavor and color changes occur during the thermal process and storage of SCM (Singh, 2004). Another physicochemical property of CM is viscosity, which increases with the addition of sugar. Crystallization of lactose is an important phenomenon in the preparation of SCM. Under the prevailing temperature and conditions in CM production, the form of aqueous alpha lactose can form crystals. The rate of lactose crystal formation is a function of the presence of colloidal substances and viscosity in the SCM (Chekalayeva & Shardun, 1982; Valle Fig ueiredo, 1980; Velez‐Ruiz & Barbosa‐Canoras, 1998).

The SCM is used to thicken creams and puddings, to create texture in ice cream, and to flavor toffees, cakes, custards, pastries, tarts, and beverages. In recent years, food industries have made significant progress in studying the effect of sucrose substitutes on various food products, which has responded to the great interest of people in consuming low‐calorie products (Mariotti & Alamprese, 2012). Fructose, also known as levulose, is a natural sweetener found in fruits at a rate of 1%–7%. The process of producing fructose syrup is that the corn starch is purified and then converted to dextrose using amylase enzymes, and finally, the syrup is produced by passing dextrose through columns containing the enzyme glucose isomerase. This syrup is considered due to its properties such as ease of metabolism, intensification of taste, thickening, stabilization, and moisture retention (Gutierrez et al., 2016; Lima et al., 2011; Wang & Wang, 2000).

Mittal and Bajwa (2012), Ramos et al., (2014) and Renhe et al., (2018) studied the sugar substitutes and thickeners to improve the organoleptic and physical characteristics of condensed dairy products. Smykov et al., (2019) studied the cooling curve in the production of SCM concentrated with whey and the effect on the size and microstructure of lactose crystals. They reported that by reducing the size of lactose crystals, the product quality was improved. The nutritional value of SCM is high in terms of fat, vitamins D, A, K, and E, essential amino acids, bone‐forming substances, and energizing sugars. Amounts of 30 to 50% vitamin B and 60 to 100% vitamin C are reduced during sterilization in evaporating milk (Kent et al., 2016).

Cinnamon (*Cinnamomum verum*) has been considered as an important food ingredient and traditional medicine since ancient times. Several reports that indicate the bioactive effects of cinnamon extract (CE) in the production of functional dairy products. Shori and Baba (2011) studied the positive effects of CE on the functional properties of bio‐yogurt. They also evaluated phenolic content, antioxidant activities of the product and inhibition of two enzymes (α‐glucosidase and α‐amylase) important in the management of type 2 diabetes. Cinnamon possesses antiulcerogenic, antiallergic, antipyretic as well as antioxidant activity (Mathew & Abraham, 2006). In addition, it contains several antioxidant compounds that can effectively inhibit the reactive oxygen species (ROS) and oxidized active molecules (H_2_O_2_, O_2_) that are produced through the formation of lipid peroxidation product and lead to cell damage, the formation of many diseases such as diabetes, inflammation, cancer, and cardiovascular diseases (Arulselvan et al., 2016; Gülçin, 2005; Mathew & Abraham, 2006; Roopha & Padmalatha, 2012; Vidanagamage et al., 2016).

Therefore, this study aimed to produce a new functional dairy product by adding CE and replacing sugar used in the formulation of SCM and evaluate the consistency and mouthfeel, particle size and stability of the product and formation of sand grains during product storage.

## 2 MATERIALS AND METHODS

### 2.1 Materials

Skim milk powder (SMP) and butter were provided by the Pegah Dairy Co. (Tehran, Iran). Maltodextrin (MD) was supplied from Sigma Aldrich (St. Louis, USA). Fructose, sucrose and cinnamon dry peel (*Cinnamomum zeylanicum*) were purchased from Tehran Grand Bazaar (Iran). All chemicals were obtained from Merck (Germany).

### 2.2 Extraction of cinnamon extract

Cinnamon dry peel was ground and passed through sieve number 40. Cinnamon extract (CE) was extracted by the cold solvent method using methanol solvent. Thus, cinnamon powder and solvent were mixed in a ratio of 1 to 10 and mixing was performed on a shaker for 24 hr at room temperature. Then, filtration was performed using filter paper with a vacuum pump and in the next stage, a centrifuge was used at a speed of 4,000 rpm for 15 min. To remove the solvent, the extracts obtained in the rotary apparatus were subjected to vacuum distillation. A vacuum of 25 mm Hg was used at 50–55ºC to minimize damage to the phenolic compounds (Jouki & Khazaei, 2010). Finally, the remaining solvent was removed with the help of nitrogen gas and the extract was stored in a dark glass container in the refrigerator (Su et al., 2007).

### 2.3 Production of SCM

The production of functional SCM was performed in a multistep process. In the first step, 20% of SMP (200 kg/t) was mixed with water at 45ºC and a thickener (0.6 kg/t) was added. Then, 8% butter was added, and a sweetener of 450 kg/t was added at 75ºC. The treatments used in this research included 7 formulations. The formulations included the replacement of fructose and the addition of CE (Table 1). The mixture was passed to a homogenizer and afterward transferred to a concentrator. After reaching the desired Brix 70, it was sent to the crystallizer and 0.3 kg/ton of lactose esterified powder was added to it at 31.5ºC and kept at this temperature for 1 hr and then cooled to 18.5ºC (Jafari et al., 2021). The samples were transferred to a filling machine, packaged and then stored at 25ºC until testing.

**TABLE 1:** Treatments used for production of SCM*

Samples	Fru: Suc (ratio)	MD (%)	CE (%)
S1 S2 S3 S4 S5 S6 S7	0:100 50:50 50:50 50:50 100.0 100.0 100.0	100 100 100 100 100 100 100	0.00 0.00 .50 1.00 0.00 .50 1.00

* MD: Maltodextrin, Fru: Fructose, Suc: Sucrose, CE: Cinnamon extract.

### 2.4 Analytical methods

To measure moisture and ash in the SCM, AOAC method was employed (Jouki et al., 2021c). The value of fat was measured by Soxhlet method (Jouki & Tab atabaei Yazdi, 2014), and protein was measured by Kjeldahl method (AOAC, 2005) by multiplying the value of nitrogen by 6.25.

### 2.5 Measurement of pH and titrable acidity

To measure the pH of the SCM samples a HANNA pH meter was used (HI 2,210, Portugal). The titrable acidity was measured according to the titrating method with 0.1 N NaOH as explained by Tayyari et al., (2017). The acidity was expressed based on lactic acid (Equation 1):

(1) Titratableacidity=(N×V×90×100)/1,000×m

where N is equal to the normality of the NaOH, V is the volume of the used NaOH, and m is the mass of the sample tested.

### 2.6 Measurement color parameters

CIE colorimeter (CR360, Minolta, Japan) was employed to measure the color parameters (L, a, and b) of the SCM samples (Jouki, 2013). The color parameters were measured as *L* (lightness), *a* (redness), and *b* (yellowness).

### 2.7 Measurement of particle size

The particle size and distribution were measured by a ZEN3600 Zeta‐sizer (MALVERN, UK) according to Dynamic light scattering (DLS) method (Jouki & Khazaei, 2021).

### 2.8 Apparent viscosity of SCM

The viscosity of SCM samples was measured by using a rotary viscometer (Brookfield DVII+Pro, USA). Briefly, 50 ml of SCM samples were poured into a container and the viscosity was measured using a spindle number 2 (LV2) at a spindle speed of 30 rpm for 30 s (Khodashenas & Jouki, 2020).

### 2.9 Functional properties of SCM

#### 2.9.1 Total phenolic content (TPC)

The TPC of SCM samples was measured using the Folin‐Ciocalteu reagent (FCR) method as explained by Milani et al., (2020). Briefly, 5 g of each SCM sample was mixed in 45 ml methanol: water solvent (80:20) using an Ultra‐Turrax homogenizer at 12,000 rpm for 2 min, and the sample was centrifuged at 10,000 × g for 15 min. Afterward, 100 μL of supernatant mixed with 200 μL FCR, and vortexed. Subsequently, 800 μL Na_2_CO_3_ (700 mmol/L) was added to the mixture and kept in the dark for 1 hr. To measure the absorption of the samples, a UV visible spectrophotometer (2100A, Unico, USA) at 765 nm was used. Results were obtained based on the curve equation of gallic acid (0.05–0.75 mM) as the standard and were expressed as mg equivalent of gallic acid (GAE) per gram of SCM.

#### 2.9.2 Antioxidant activity

The DPPH method was employed to determine the antioxidant activity of SCM samples. Briefly, 5 g of each SCM sample was mixed in 45 ml methanol: water solvent mixture (80:20). The mixture was centrifuged at 5,000 rpm for 10 min, and 100 μL of the supernatant was added to 3.9 ml of 0.1 mM methanolic DPPH solution. The solutions were kept in the dark box for half an hour, and the absorbance was determined at 517 nm (Jouki et al., 2021a), and the radical scavenging activity for each SCM sample was calculated by Equation 2:

(2) $$\mathrm{Antioxidant}\;\mathrm{activity}\;\left(\%\right)=\frac{{\mathrm{Abs}}_{\mathrm{DPPH}}{‐\;\mathrm{Abs}}_{\mathrm{sample}}\;}{{\mathrm{Abs}}_{\mathrm{DPPH}}}\;\times 100$$

#### 2.9.3 Scanning electron microscopy of microstructure of SCM

To study the microstructure of the SCM samples a scanning electron microscope (AIS‐2300, Seron Technologies Inc., Gyeonggi‐do, Korea) was used. Firstly, the SCM samples were freeze‐dried using a vacuum freeze dryer (VaCo 5‐D, Zirbus, Germany) and mounted on holders and covered with gold in a vacuum sputter coater. Then, electron scanning was conducted using a beam accelerator at a voltage of 5.0 kV for the external surface (Alipoorfard et al., 2020).

#### 2.9.4 Sensory evaluation

To evaluate the sensory properties of the SCM samples, a 5‐point hedonic test was selected. The SCM samples were given to 9 trained panelists, including 5 women and 4 men. Hedonic test score (5 = like extremely, 4 = like a little, 3 = neither like nor a dislike, 2 = a little dislike and 1 = dislike extremely) for taste, odor, color, texture, and overall acceptance by the board expert panelists (Shariati et al., 2020).

#### 2.9.5 Statistical analysis

The statistical analysis was performed through the subjection of data to the analysis of variance (ANOVA) by SPSS software (version: 22.0, SPSS Inc.) and Duncan multiple comparison tests were employed to determine the differences between the means (*p* <.05).

## 3 RESULTS AND DISCUSSION

### 3.1 Chemical composition of SCM

The moisture content, fat, protein, and total dry matter in the SCM sample were 27.6, 8, 8.43, and 72.4%, respectively. Similar results have been reported in other studies regarding the percentage of chemical compounds in SCM. Renhe et al., (2018) reported that the average total solids in the sweetened condensed milk were 72.59%, resulting in moisture content of 27.41%. Ahmed et al., (2019) reported that the moisture, fat, protein, and dry matter levels of sweetened condensed milk samples were 19.9, 19.8, 7.95, and 80.1%, respectively. Protein is one of the most important compounds in the process that has largely determined the thermal stability and viscosity of condensed milk in physicochemical reactions (Silveira et al., 2015). Moisture content is a key factor in creating the strength and stability of the structure of condensed dairy products (Gharibzahedi et al., 2012; Jouki & Khazaei, 2013). These features play an important role in determining the process efficiency, final aspects of evaporation, water activity and lactose crystallization rate (Jouki et al., 2021b; Renhe et al., 2018). Based on the findings of Renhe et al., (2018), the maximum level of moisture content of sweetened condensed milk is 30% (w/w). Therefore, according to the results obtained from this study, the moisture and dry matter values of the SCM samples were in accordance with previous research and within acceptable limits.

### 3.2 Acidity and pH of SCM

**FIGURE 1:**
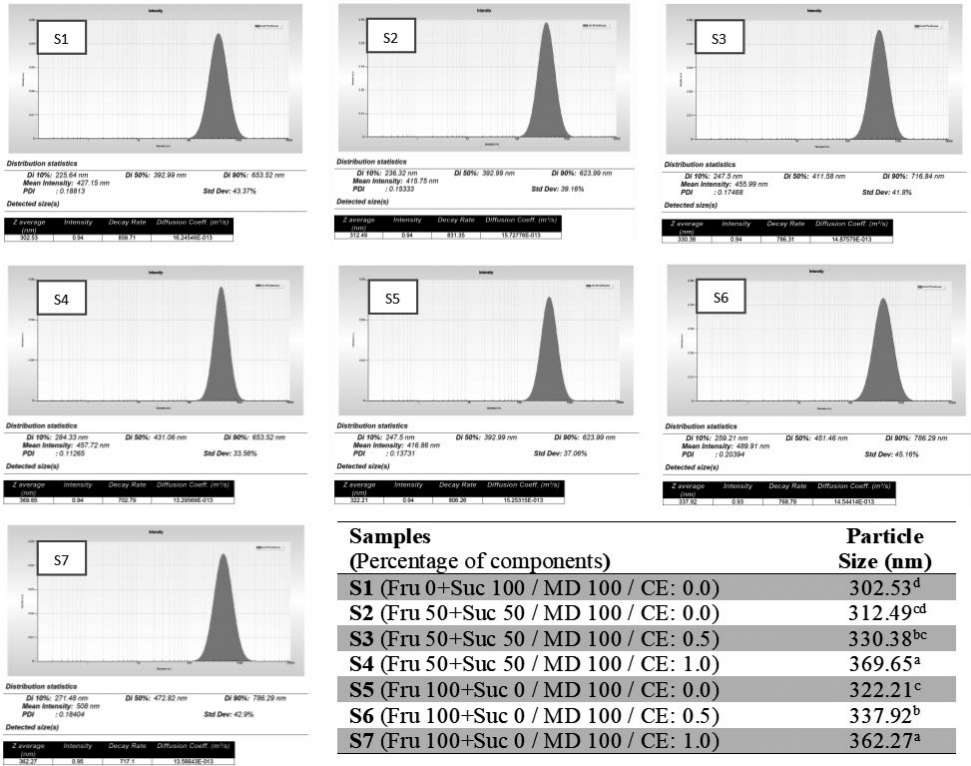
Changes in pH, acidity, and viscosity of the SCM samples during storage time

As Figure 1a shows, there was a significant difference between the acidity of formulated SCM samples and the control (*p* <.05). The acidity of the SCM samples did not change significantly (*p* ≥.05) during the storage time. Replacement of fructose increased the acidity of SCM samples (*p* <.05). The highest level of acidity was observed in the samples containing maltodextrin, fructose and lactose and cinnamon extract (S7), and the lowest level of acidity was observed in the control sample (S1). Figure 1a also shows that the acidity of the SCM samples containing maximum levels of fructose and cinnamon extract (S7) increased significantly during storage, and at the end of the storage time, these samples showed the highest acidity and lowest pH value.

As Figure 1b shows, the highest pH was observed in the control sample (S1), and the lowest pH was seen in the S7 sample. These results were consistent with the results obtained on the acidity of the SCM samples. As stated by Azarnia et al., (1997) and Khazaei et al., (2011), one of the effective factors in reducing pH and increasing acidity is the increase in humidity due to the presence of hydrophilic compounds, which is followed by intensified lipolysis. Moreover, the production of fatty acids as well as the complete conversion of lactose to lactic acid during storage can also be effective factors (Azarnia et al., 1997; Jouki & Khazaei, 2012). As Cerníková et al., (2008) reported, the level of acidity decreases with increasing the amount of carrageenan in processed cheese. According to the results of this study, by reducing sucrose in the SCM formulation, the acidity increased and as a result, the pH of the decreased.

Alizadeh et al., (2014) and Raftani Amiri et al. (2013) observed significant changes in pH with increasing the percentage of stevia and maltodextrin in a fruit‐based milkshake and yogurt formulation, respectively. As Figure 1b shows, the decrease in pH values on the last day of storage was greater than on the first day. As reported by Raftani Amiri et al. (2013), milk concentration and increased hydrolase enzyme activity have led to a decrease in pH. Therefore, lowering the pH of SCM by increasing the replacement of fructose may be related to the nature and structure of this compounds, which reduce pH and increase acidity by changing the viscosity, water holding capacity, and enzymatic activity (Alizadeh et al., 2014; Haji Ghafarloo et al., 2020; Raftani et al. 2013).

The effect of adding cinnamon extract on reducing the pH and increasing the acidity during the storage period was also statistically significant (*P* ˂ 0.05). Similar results of increasing acidity and decreasing pH due to the addition of plant extracts in other dairy products have been reported (Patil et al., 2017; Singo & Beswa, 2019). As reported by Singo and Beswa (2019), the addition of roselle extract increased the acidity of ice cream. In addition, Patil et al., (2017) showed that the addition of the mint (*Mentha arvensis)* extract at a level of 0.5%–1.5% to the ice cream formulation increased the acidity from 0.27 to 0.35, which might be due to the presence of phenolic compounds in mint extract that increased the acidity of the product.

### 3.3 Viscosity of SCM

As it is shown in Figure 1c, the viscosity of SCM samples was in the range of 560 to 800 mPa.s, which was in agreement with the reports of Ahmed et al., (2019) and Renhe et al., (2018). The changes in viscosity of SCM samples during the storage time are shown in Figure 1c. There is a significant difference between the treatments and the control sample (*p* <.05). Also, the viscosity increased over time that was statistically significant (*p* <.05). As Figure 1c shows, sugar replacement decreased the viscosity of SCM (*p* <.05). As has been reported by Koksoy and Kilic (2004), and Paraskevopoulou et al., (2003), the viscosity of dairy products is changed in presence of gums.

The addition of cinnamon extract also reduced the viscosity of the SCM samples (*p* <.05). Similar results have been reported in other studies on the effect of plant extracts on the viscosity of dairy products (Haji Ghafarloo et al., 2020; Shariati et al., 2020). Haji Ghafarloo et al., (2020) reported that the lowest viscosity of dairy drink was related to the sample congaing 0.5% ginger extract. As they stated the addition of herbal extract to the product formulation can cause a decrease in viscosity and consistency of the dairy product. The highest viscosity was seen in the control samples (S1), and the lowest viscosity was seen in the S7 samples (100% fructose and 1% CE).

As Anema et al. (2004) showed, changes in viscosity of dairy products can be related to changes in pH, acidity, particle size, and protein deposition in dairy products during storage. They stated that these changes could be due to the whey protein‐casein interaction that is also dependent on pH changes. They also showed that parameters such as particle size and protein deposition linearly lead to changes in the viscosity of dairy products during the storage period. In general, with increasing the value of fructose, the viscosity of the SCM samples decreased, which can be attributed to the lower viscosity created by the equal concentration of fructose than sucrose. Telis et al., (2007) reported that the viscosity of sucrose and fructose solutions at 50% concentration at 25ºC was 11.49 and 8.00 mPa.s, respectively. In addition, José Montañez‐Soto et al., (2013) studied the rheological properties of different syrups. They found that fructose syrup has a lower viscosity (170 mPa.s) than sucrose syrup (250 mPa.s), which can explain the lower viscosity of fructose‐formulated SCM than samples containing sucrose.

The results of our study were in agreement with previous studies. As stated by Guggisberg et al., (2011), the viscosity of yogurt increased with increasing the stevia concentration. In addition, Lisak et al., (2012) showed that the viscosity of strawberry yogurt increased by adding stevia. However, Homayouni Rad et al. (2012) demonstrated that the viscosity of cocoa milk samples containing 100% stevia and 6% inulin was higher than control SCM samples. The reason for the discrepancy in the reported results could be related to stevia‐related hydrocolloids to compensate for tissue weakening due to sugar replacement, differences in product type and type of additives. Also, the viscosity of the SCM samples increased significantly during the storage period, which could be due to partial deposition and phase separation in the SCM samples.

### 3.4 Particle size of SCM samples

**FIGURE 2:**
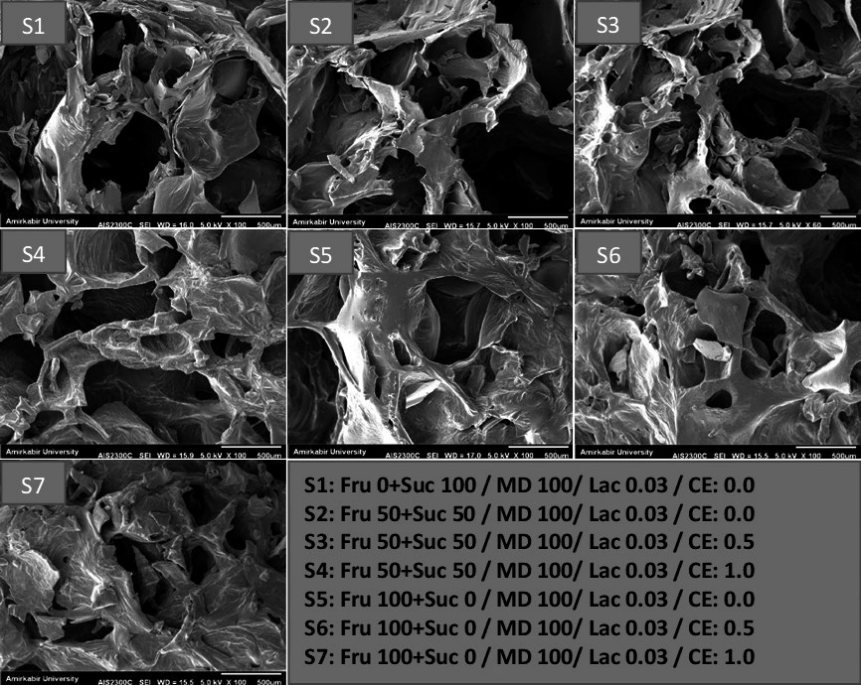
Particle size of SCM samples

Figure 2 shows the particle size results of SCM samples. There is a significant difference between the formulated SCM and the control sample (*p* <.05). Particle size increased with increasing fructose levels (*p* <.05). As stated by Cornillon and Salim (2000), the product texture can be affected by the content and distribution of moisture in low‐moisture food products. In addition, Rosell et al., (2001) stated that water absorption and consequently particle size increases due to the formation of hydrogen bonds and the presence of a large number of OH groups in the food. In this study, maltodextrin was used to create concentration and stability in the product, which can absorb water and form hydrogen bonds with water.

Zheng et al., (2007) and Huda et al. (2003) found that maltodextrin has water‐friendly properties, and the reason for the increase in moisture absorption in compounds with a high proportion of maltodextrin is the presence of low molecular weight sugars in the compounds of these substances. The higher the hydrophilicity of the compounds, the higher the water uptake rate and consequently the particle size increases. As stated by Silva et al., (2020b), the OH‐treated Dulce de leche presented higher numbers of lactose crystals of smaller size. The results of particle size analysis show that the addition of the CE at concentrations of 0.5 and 1% increased the particle diameter in SCM. Increasing the particle diameter can be related to increasing the dry matter and creating more hydrogen bonds in the product by adding the CE.

### 3.5 Functional properties of SCM samples

#### 3.5.1 Total phenolic content (TPC) and Antioxidant activity

The TPC and antioxidant activity of pure CE, control SCM, and SCM containing CE are shown in Table 2. The TPC and the DPPH free radical scavenging activity for CE were 417.45 μg GAE ml^‐1^ and 80.12%, respectively, which was consistent with the results of previous research by Khawsud et al., (2020) and Vidanagamagea et al. (2016). Khawsud et al., (2020) found that the PC and the DPPH free radical scavenging activity for CE were 428.41 ± 4.59 μg GAE ml^‐1^ and 81.33 ± 3.22%. In addition, Khawsud et al., (2020) reported values of 656.91 μg GAE ml^‐1^ and 87.31% for TPC and antioxidant activity of the cinnamon extract, respectively. The TPC and antioxidant activity of the control and formulated SCM samples were in the range of 29.29–35.40 μg GAE ml^‐1^ and 8.32%–9.13%, respectively. These results were in line with the findings reported by Shori and Baba (2011). They reported that the TPC and antioxidant activity in bio‐yogurt was 35.3 μg GAE ml^‐1^ and 26.4%, respectively.

**TABLE 2:** TPC and antioxidant activity of CE and SCM samples*

Samples	TPC (μg GAE ml^−1^)	DPPH activity (%)
CE (Pure cinnamon extract) S1 (Control SCM) S2 (SCM Formulated with 50% Fru+0.0%CE) S3 (SCM Formulated with 50% Fru+0.5%CE) S4 (SCM Formulated with 50% Fru+1.0%CE) S5 (SCM Formulated with 100% Fru+0.0%CE) S6 (SCM Formulated with 100% Fru+0.5%CE) S7 (SCM Formulated with 100% Fru+1.0%CE)	417.45 ± 4.59^a^ 32.23 ± 2.34^d^ 35.40 ± 3.87^d^ 143.24 ± 5.30^c^ 265.78 ± 6.30^b^ 29.29 ± 3.76^d^ 139.21 ± 3.44^c^ 249.29 ± 6.93^b^	80.12 ± 3.22^a^ 9.13 ± 1.42^d^ 9.02 ± 1.21d 50.12 ± 2.10^c^ 66.45 ± 2.56^b^ 8.32 ± 1.85^d^ 52.02 ± 2.49^c^ 62.92 ± 3.20^b^

* Values are the mean±*SD*; values with different lowercase superscript letters in the same column are different (*p* <.05).

The addition of CE in concentrations of 0.5 and 1% to the SCM formulation increases the TPC and free radical scavenging activity in the SCM samples up to 139.21–143.24, and 249–29–265.78 μg GAE ml^‐1^, and 50.12–52.01 and 62–92%–66.45%, respectively. Shori and Baba (2011) investigated the effects of CE on the antioxidant activity, total phenols, proteolysis, and inhibition of α‐amylase and α‐glucosidase in bio‐yogurts prepared from cow and camel milk and produced a functional dairy product. They stated that the addition of cinnamon extract in bio‐yogurts increased the total phenols and antioxidant activity of this dairy product throughout 3 weeks of storage at 4ºC. Khawsud et al., (2020) showed that the TPC and antioxidant activities of the reduced‐fat ice cream incorporated with cinnamon powder at 0.1% was significantly higher than control samples (*p* <.05). Therefore, these findings showed that CE with high levels of TPC could lead to increased functional properties of SCM.

### 3.6 Microstructure of SCM

**FIGURE 3:**
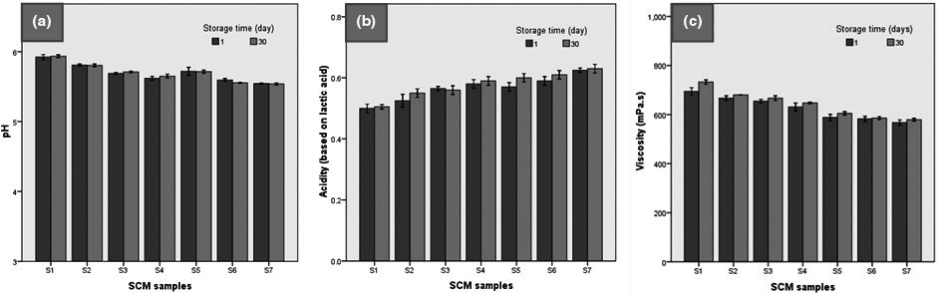
Microstructure of SCM samples

Figure 3 shows the electron microscope images of the microstructure of SCM samples. As can be seen, the control sample (S1) had a sheet texture and a large number of formed cavities, but the diameter of the cavities was smaller than the other samples and the formed cavities had irregular shapes. In the presence of 50% fructose (S2–S4), the cavities became bigger but had also a sheet texture and a large number of cavities as well as irregular and elongated shapes. Thus, the formation of a three‐dimensional network to trap air and gases in condensed milk tissue is associated with an increase in stiffness due to an increase in the number of bonds formed by maltodextrin and fructose with milk proteins and a reduction in cavities in the product tissue.

The image obtained from the S5–S7 samples is similar to S2–S4 samples except that the number of cavities decreases and the diameter increases. The number of cavities has increased and the interconnectedness of the tissue has decreased. As Figure 3 shows, with increasing the percentage of fructose replacement in SCM, the number of tissue cavities decreased. Fructose plays an important role in tissue hardness because the smaller the bubbles and cavities in milk tissue, the harder it becomes.

In this regard, Sahraiyan et al. (2014) showed that when the number of cavities in the products increases, and these small cavities are evenly distributed in the sample, increasing the pasteurization temperature cannot lead to excessive expansion and their rupture. Otherwise, with the weak strength of the cavity walls, due to the expansion of the cavities, the structure of the product will weaken and collapse rapidly. The electron microscope images show that by adding the CE to the SCM formulation, the number of cavities is reduced along with their size. Adding the extract increases the dry matter of the product and more bonds with water molecules and compounds in milk cause more cohesion of the structure and more concentration of the product.

### 3.7 Color parameters of SCM

**FIGURE 4:**
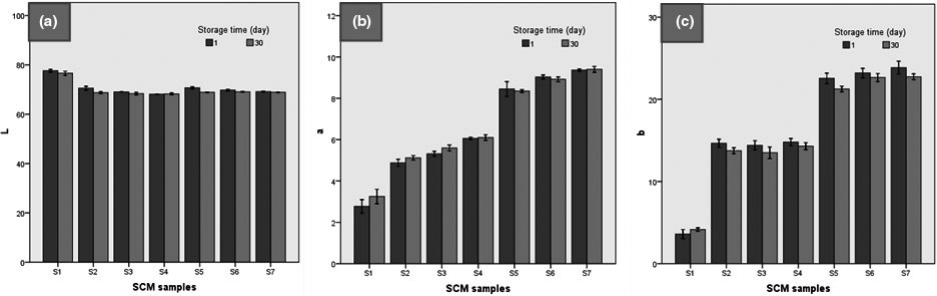
Color parameters (L, a, and b) of SCM samples during storage time

Figure 4 shows the changes in color parameters (L, a, and b) of SCM samples by adding extract and replacing sugar. The L (lightness) value of SCM decreased with increasing the fructose replacement (*p* <.05). The addition of the CE reduced the lightness of SCM samples. This result can be due to the increase in dry matter and further concentration of the samples and thus reduce the reflection of light by them. Similar results have been reported by other researchers in reducing the lightness of dairy products by adding plant extracts (Khawsud et al., 2020; Yeon et al., 2017).

As Figure 4 shows, with increasing the level of fructose replacement, the redness and yellowness increased (*p* <.05). These results were consistent with the findings of Amerinasab et al., (2015). They reported that as the concentration of date sugar in yogurt increased, the color parameters of yellowness and redness increased. In another study, Jridi et al., (2015) reported that the addition of date syrup to dairy desserts increases the color parameters of the dairy product. The interaction between existing reducing sugars and milk proteins occurs during the Maillard, which may lead to the formation of color compounds in the product and by increasing its amount in the formulation of SCM the color indicators increase.

The lightness value was highest in the order of S1˃ S5˃ S2 ˃S3=S6 > S4 ˃S7, and the redness (a) value was highest in the S7 sample (SCM formulated with 100% fructose, and %1 CE), which was considered to be an effect of the light brown color of the CE. Although the b (yellowness) value of the samples containing 0.5% CE did not change compared to the control, the samples containing 1% CE had the highest amount of yellowness among the samples (*p* <.05). As stated by Khawsud et al., (2020), the decrease of L and increase in a and b of dairy products related to the decreasing of whiteness. In addition, Yeon et al., (2017) reported that by adding pepper powder to ice cream, the L value decreased and the values of a and b increased. Figure 4 also demonstrated that the values for L and b decreased during the storage period (*p* <.05), while the redness did not change significantly (*p* ≥.05). In agreement with our results, Peker and Arslan (2017) found that the lightness and yellowness values of yogurt were decreased during storage time.

### 3.8 Sensory properties of SCM

**FIGURE 5:**
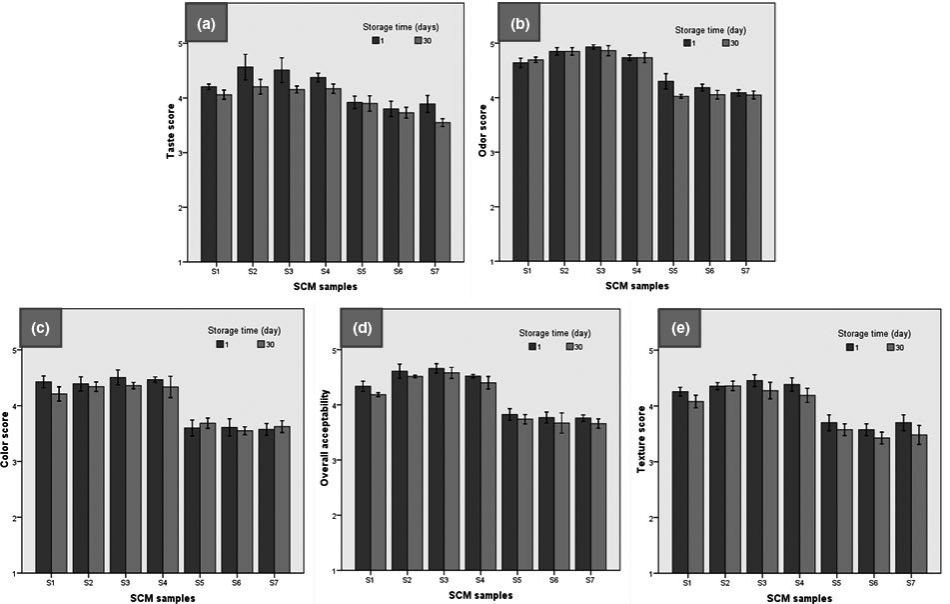
Sensory properties of SCM samples during storage time

Figure 5 Shows the sensory attributes in SCM samples throughout the storage time. There was a significant difference between treatments in terms of taste score (*p* ˂0.05). It can be inferred that the application of fructose significantly increased the taste score of SCM the samples (*p* ˂0.05). Fontvieille et al., (1989) showed that fructose has more benefits than sucrose, including a lower increase in plasma glucose and insulin response, and a sweeter taste. Therefore, the higher taste scores of SCM samples in which sucrose has been replaced with fructose may be related to the higher sweetness of the fructose and its more suitable taste. Silva, Rocha, Guimarães, Balthazar, Scudino, et al., (2020) studied the effects of ohmic heating (OH) on the sensory properties of Dulce de leche (sweetened condensed milk). They used a 9‐point hedonic scale test and reported some of Dulce de leche's sensory characteristics, including appearance, aroma, taste, texture, and overall acceptability. They showed that ohmic heating treatment could be effective in improving the sensory acceptance of this dairy product.

The SCM samples containing 50% fructose as a sugar substitute and maltodextrin (S2–S4) had the highest taste scores. The results of the sensory test also showed that the panelists gave higher scores to the samples containing 50% fructose replacement than the samples containing 100% fructose. This suggests that due to the higher sweetness of fructose than sucrose, the substitution of 100% sugar can produce excessive and unsuitable sweetness in terms of taste. Also, the use of fructose along with maltodextrin and CE in the SCM formulation increased the odor score (*p* ˂0.05). In this case, S3 sample received the highest score from the panelists (Figure 5b). These results were also observed at the end of the storage period and S3 sample received the highest score (*p* ˂0.05).

The use of fructose significantly increased the color score of the SCM samples (*p* ˂0.05) (Figure 5c). On the first day, the higher color scores were related to the control, and the samples containing 50% fructose and the lower scores were seen for the samples enriched with 100% fructose. On the first day and during the 30‐day storage period, S2–S4 samples had the highest color and overall acceptability scores. These results show that replacing 50% sucrose with fructose and using maltodextrin, in addition to the use of 0.5% CE, lead to the production of a product with desirable sensory attributes. Due to the desirability and popularity of cinnamon and compatibility with the taste and odor of dairy products, samples containing cinnamon extract in low concentration (0.5%), which contained maltodextrin and 50% fructose replacement, received the highest taste, odor, and overall acceptability scores.

The results of our study were consistent with previous reports in terms of replacing sucrose with other sweeteners in the production of SCM. Shimoda et al., (2001) studied the effect of flavored compounds on the properties of SCM. They stated that the volatile flavor compounds promoted the sensory scores of SCM. In addition, Lee et al., (2005) showed that by replacing sucrose with a mixture of sucralose and maltodextrin in amounts of 60 and 80%, the color of the cake crust becomes lighter. They showed that the application of sucralose and maltodextrin enhances the sensory attributes of the product.

As Ghandehari Yazdi et al. (2014) showed, replacing sucrose with a mixture of sucralose and maltodextrin makes milk desserts brighter. Decreased browning index due to the replacement of sucrose with diet sweeteners on a variety of desserts has been reported in many studies (Cardoso and Bolimi 2007; Mittal & Bajwa, 2012; Renhe et al., 2018). Mittal and Bajwa (2012) found that a 4% substitution of inulin in flavored milk drinks was effective in flavoring and texturing the product and improved the sensory attributes.

## 4 CONCLUSION

In this study, the effect of using cinnamon extract and fructose substitution in SCM formulation in the production and characterization of the flavored dairy product was investigated. Physicochemical, functional, and sensory properties of the product such as pH, acidity, color, viscosity, total phenols, antioxidant activity, microstructure, and sensory attitudes of the SCM samples were determined during storage. Replacing sucrose with fructose increased acidity, redness and yellowness, and sensory scores, and decreased viscosity and particle size of SCM samples. During storage time, the pH, lightness (L), and yellowness (b) of SCM samples decreased and the viscosity and redness (a) increased. Although fructose replacement increased particle size, maltodextrin prevented an increase in particle diameter and stabilized SCM samples. The addition of CE with antioxidant properties to the SCM formulation and replacement of sugar resulted in the production of a stable and functional dairy product. Finally, based on physicochemical, functional and sensory tests, SCM sample formulated with 0.5% CE, and replacement level of 50% fructose was introduced as the desired sample. This formulation can be used in the dairy industry to produce flavored functional dairy product.

## CONFLICTS OF INTEREST

The authors have declared no conflicts of interest for this article.

## AUTHOR CONTRIBUTIONS

**Mohammad Jouki:** Data curation (equal); Funding acquisition (equal); Investigation (equal); Project administration (equal); Writing‐review & editing (equal). **Somayeh Jafari:** Formal analysis (supporting); Writing‐original draft (equal). **Ali Jouki:** Formal analysis (equal); Software (equal). **Naimeh Khazaei:** Formal analysis (equal); Software (equal); Writing‐original draft (equal).

## ETHICAL APPROVAL

This study does not involve any human or animal testing.

## Data Availability

Duncan's multiple comparison test is used as a common method to compare the average effects of treatments used in the food process. The authors investigated the physicochemical, functional, and sensory properties of SCM samples during the storage time using SPSS VER: 22 software.
